# Storying COVID-19: fear, digitalisation, and the transformational potential of storytelling

**DOI:** 10.1007/s11625-021-01031-9

**Published:** 2021-09-06

**Authors:** Maja Essebo

**Affiliations:** 1 Lund University Centre for Sustainability Science (LUCSUS), Biskopsgatan 5, 223 62 Lund, Sweden; 2 Lund, Sweden

**Keywords:** Sustainability science, COVID-19, Narrative theory, Story, Climate change

## Abstract

Stories are being increasingly recognised for their potential as creators, not only depicters, of change. As such, they are receiving greater interest within sustainability science, not least in the approaches specifically focused on transformative processes of co-creation. But while highly powerful, stories are confined by both inherent and external frameworks that, if not acknowledged, limit their transformative potential. This paper addresses two such critical issues—fear and digitalisation—and discusses the ways in which they influence how and with what effects stories can be told. It uses the COVID-19 pandemic as illustration of storytelling processes and outlines some of the ways in which we can, and cannot, draw parallels between pandemic and climate change storytelling.

## Introduction

Crises are amazing story proliferators. We tell tales of the crises that have been, of the crises yet to come, and most of all of the crises unfolding in the here and now. In trying to braid rapidly evolving global events with our personal lives and experiences, stories are equally a means of communication as they are coping mechanisms (Malinowski 1954). Stories are also recognised for their potential to influence, even change, events, including practices, beliefs, and materialities, through processes of sense-making and creating shared convictions and desires (Arlow 1961). As such, their role is to imagine a reality more than to describe it (Tismaneanu 1998), making them potentially highly constructive incitements for change as they guide small, everyday decisions and practices to line up with their narrative sense.

Holding this potential for change, stories have become of considerable interest to sustainability science research (Brown 2017; Paschen and Ison 2014; Veland et al. 2018). Not least in relation to processes of transformation, understood as deliberate and fundamental reordering of human and environmental interactions (Braun 2015; O’Brien 2012; Westley et al. 2011) which create genuinely alternative futures (Kates et al. 2012). Creating an alternative future requires an act of imagination, of envisioning that which is not. Here, the role of stories has been explored in various forms and practices, including identifying and evaluating different, and potentially clashing, sustainability narratives (e.g. Bremer and Funtowicz 2015; Frank 2017; Luederitz et al. 2017); constructive and co-creative approaches which often centre on notions and (re)creations of ‘the good life’ (e.g. Bliss and Fisher 2014; Garnett 2014; O’Neill et al. 2018a), using storytelling for communicating science to and with a broader audience (e.g. Dahlstrom 2014; Martinez-Conde and Macknik 2017), and, more recently, the exploration of using arts as means for understanding, telling, and possibly altering narratives of sustainability (e.g. Heinrichs and Kagan 2019; Nakagawa and Saijo 2021; Pröpper 2017).

Storytelling does hold vast transformative potential, yet dealing with stories is a complex and messy affair. This paper argues that the continued advancement of narrative approaches within sustainability science requires a more thorough engagement with both the story’s elements—plot, time, fear/hope, and character creation—and with its mediums. To clarify these points, the paper will discuss fear (element) and digitalisation (medium), using the COVID-19 pandemic as illustration. In the discussion section, it will draw parallels to climate change storytelling, showing how superficial similarities, i.e. global crises requiring immediate and extensive action, can hide vital differences. These differences emphasise the need for a more fundamental understanding of the complexity of story and storytelling.

The paper starts with a brief presentation of the concept of story and some of its most crucial elements: plot, time, and character creation. This section also includes a discussion on the role of story in sustainability science and transformational storytelling. Second, the paper discusses the issues of fear and digitalisation and how they affect the transformative potential of storytelling. These are both illustrated using COVID-19 storytelling, which, in the last section, is discussed alongside climate change storytelling with the purpose of showing how crucial differences caution us against one-to-one comparisons. Ultimately, the aim of the paper is to engage with the use of narrative approaches in sustainability science by identifying how intrinsic story elements as well as technical contexts affect the potential for transformational storytelling.

## Conceptual framework: story & transformation

### Story

It is vital to underline that the stories discussed in this paper are not of the once-upon-a-time variety, but rather everyday stories that help make sense of our experiences and beliefs. These are often fragmented pieces, functional only in their connection to other fragments, people, places, or events. As oppose to meta-narratives or canonical storytelling, these are the ‘small stories’ (Bamberg 2006; Bamberg and Georgakopoulou 2008; Georgakopoulou 2006) that make up a large part of our daily interaction, e.g. chatting with a co-worker or sending a text to a friend. As such, they are part of cognitive structures representing and making sense of the everyday world around us and our place in it (Bruner 1990). To tell a story is to relate events in a narrative form (Ingold 2016). Importantly, past events and present events all belong to the same yarn as there is ‘no point at which the story ends and life begins.’ (Ingold 2016, p. 93). Another word for Ingold’s yarn is plot and it is one of the distinguishing features of story (Herman 2004).

In its most essential interpretation, a plot can be considered as a line of contingencies that move the story along (Ricoeur 1980). It weaves together characters and events in time and space, a process known as emplotment (White 1973), leaving us with a sense of completeness. Plot does not always make sense as it unfolds, but must do so in hindsight. However, ‘sense’ should never be confused with ‘facts’ or ‘truth’ as sense here refers to a narrative sense, a subjective sense that we can share with others through storytelling. One should also note that while time, or events unfolding over time, is central to plot, this is not to say that representation of time equals the actuality of time. Deleuze likes to say that ‘time is out of joint’ (Deleuze 2004) while Serres likens time to ‘a crumpled handkerchief’ (Serres 1995). Similar notions have been put forward by Latour, Tarde, Whitehead, and Bergson, all of whom emphasise non-linearity, flow, and disruption. Still, while intellectually fruitful and far more honest to the nature of existence, ‘it’s a mess’ is not terribly helpful in understanding everyday experience. This is where storytelling can help, in *re-presenting* time as the linear unfolding of one event leading to the next. In many ways, we can think of a story as a narrative act, and as the outcome of a narrative act, that turns chaos into order. Story frames events in the terminology of a beginning, a middle and an end, a characteristic which may seem simplistic, but that can be highly useful, not least in determining what is *not* a story.

Plot is closely related to characters, or what narratologists, such as Greimas (1987) and Bal call ‘actants’ (Bal 2009). The characters—agents, actants—experience and drive the plot forward through interaction with other characters, events, or with their own thoughts and experiences and help to create understanding through their being, their *characteristics*. The way they act is interpreted through what we know about them, or about ourselves in the case of first-person storytelling which is arguably the most common kind. Actions, preferences, or choices are explained and understood through contextual and cultural circumstance and shared understanding, e.g. ‘of course Mary lied, she’s a politician’ as compared to ‘of course Mary likes blue, she’s a carpenter’. Exceptions are uncommon and uncomfortable—out of character—as characters acting in ways that are not aligned with our perceptions can lead to a suspension of disbelief (Bates 1994), which in fiction boils down to them no longer being perceived of as ‘credible’. In real life, however, such character missteps cannot be explained by author oversight, but will require that we either (i) ignore the exception, (ii) find alternative ways of aligning actions with pre-conceived notions, or (iii) re-evaluate our own understanding. The first of these three is undeniably the simplest while the second and third require constructive and introspective effort. They are also the basis for change and, as such, of vast interest for theories and approaches centred on transformation. This emphasises the prescriptive aspect of story, that in telling the world we form it and that in order to change it, we need to ‘tell it differently’.

### Transformational storytelling

The use of stories, or storytelling, in transition and transformation research focuses on its constructive aspects, specifically its role in creating practices and materialities that cannot be challenged by facts alone, but need to be met on their own narrative terms. Included in this field is a growing number of sustainability science researchers who, possibly due to the contrast between overwhelming evidence and actual change and supported by the transdisciplinary characteristic of sustainability science, put hope into the power of narrative-induced change. Here, we find the growing interest in finding and in some cases even creating stories capable of ‘telling things into being’. In this approach, the potential of storytelling is considered both from a methodological perspective, where transformative or disruptive events are understood through narrative research strategies (e.g. Hards 2012), and as a transformative action in itself through positive re-framing (e.g. Sharp 2018). These views resonate in the emancipatory approach (Butler 1990), arguing for change through performativity, i.e. re-shaping identities and actions by choosing certain performances over others. The notion of performativity both refutes (metaphysical) presumptions and makes ontological claims in that it ‘starts to describe a set of processes that (…) bring into being certain kinds of realities’ (Butler 2010, p. 147). These performatives, in the form of speech acts, are a more nuanced form of what Austin (1962) characterised as perlocutionary; speech acts that that through a specific force and with certain conditions in place can come into being through the action of the audience, e.g. a superior telling its staff that camaraderie is a vital part of the work culture leading to employees instituting Friday coffees.

Within sustainability science, these approaches can be found in the concept and practice of co-creation which aims to integrate knowledge between academic fields as well as between researchers and societal actors to conduct solution-oriented research and practice (Brandt et al. 2013; Mauser et al. 2013). Ultimately, these approaches aim to not only analyse, but encourage change, e.g. Wolgemuth and Donohue (2006) who draw on Megan Boler’s pedagogy of discomfort (Boler 1999) to propose a narrative research method they term ‘an inquiry of discomfort’, aimed at ‘transforming participants’ lives by opening up new subjective possibilities’ (Wolgemuth and Donohue 2006, p. 1024). Rather than fighting fiction with facts, these approaches accept and value the positive forces of storytelling. Narratives that can be construed as harmful are made visible, but not rejected as ‘nonsense’ but rather met with alternative storytelling through processes of co-creation. Such approaches also include imaginative scenario creation (Pereira et al. 2019), creative non-fiction (Löschnigg and Braunecker 2019), and alternative histories (De Cock et al. 2019).

Many such works draw on variations of ‘the good life’, the fundamental idea of which is to (re)construct notions of what the good life *could be* in order to harness pathways of positive change towards alternative and sustainable practices (e.g. Di Giulio and Rico 2019; O’Neill et al. 2018b; Schmidt 2018). These have been addressed as anything from specific methodological tools designed to understand underlying values and community development (e.g. Bliss and Fisher 2014; Garnett 2014) to overarching, interdisciplinary re-imaginations (e.g. Syse and Mueller 2015).

These approaches are valid, constructive, and, not least, essential to actively engage with processes and practices of change. But in order to do so, we must also recognise that storytelling is complicated affairs made to look simple, i.e. packaging the intrinsically byzantine relationships, knowledges, histories, and practices of human interaction in neat narrative parcels. If sustainability science is to engage with the transformative potential of storytelling on a deep and constructive level, the messiness of narrative practices needs to be better acknowledged. The next section addresses two complicating factors to illustrate how stories need to be understood not only as wholes, but as parts or elements, illustrated here by the element of fear, and as results of not only content, but of form and technology, in this case that of digitalisation.

### Fear & digitalisation in COVID-19 storytelling

In addressing the issues of fear and digitalisation, the paper will illustrate its points by applying them on the COVID-19 pandemic. The nature and span of the COVID-19 pandemic has acted as a veritable Petri dish of storytelling. Many of these stories cross over into the bizarre, such as the virus being spread via 5G towers (Tchéhouali 2020) or Bill Gates using a vaccine to implant people with microchips (Brown and Weiseusa 2020), but others reside on that fine line between fact and fiction that make them both easily adoptable and hard to shake. These include various origin stories, such as SARS-CoV2 being bioengineered in a Wuhan laboratory, which has experienced a revitalisation of late, or the many treatment stories said to either prevent or reduce the risk of contracting COVID-19.

The story examples are chosen partly due to their exemplifying qualities, i.e. their ability to clarify a point, and partly due to their proliferation, i.e. an appreciation of them having achieved certain spread and general acceptance and/or awareness. They are not intended as case studies, but as illustrations of the case in point. As such, this paper is taking a kaleidoscopic view rather than a telescopic.

### Fear

The use of fear, as either a motivator or dampener of change, is greatly debated. Its infectious nature along with its strong emotional impact would make it a strong candidate for emotionally induced action, yet using fear appeals to motivate behavioural changes, e.g. in response to health risks (Covello et al. 1988), often fails. Similar tendencies are shown in responses to sustainability threats, where ‘sustainability or collapse’ narratives serve to induce apathy and non-action (Strunz et al. 2019). One of the deciding factors in fear response is efficacy, i.e. the ability to produce the intended result. Strong fear appeals with high efficacy lead the largest response while strong fear appeals with low efficacy tend to lead to defensive responses (Witte and Allen 2000). In facing threats that arouse a great deal of fear, we need clear notions of how to address it lest we become wary, distrustful, and dismissive. Here, storytelling plays a crucial role related to one of its central uses, simplification.

In truth, plotting any type of human practice or belief on a y to z grid, the dots would be scattered, constantly shifting, and ethically, emotionally, and logically contradictory. Stories, however, rarely show such complexity but rather works in absolutes. Yet, it is important to note that the intent is not to hide complexity, but to deal with it through what Malinowski (1954) calls ‘the psychological factor’; relieving anxiety by creating narrative frameworks that help create order, understanding, and a narrative course of action (or non-action). Similarly, Cassirer (1946) suggests that by naming our fears, we not only label them but also identify ways of avoiding them. What we are naming, here, is good and evil, right and wrong, protagonist and antagonist. Lévi-Strauss goes as far as to say that classification in terms of opposites is an inherently human trait (Lévi-Strauss 1978). In naming the villain, we identify both our opponent and the way in which we can overcome it, whether the villain is a physical representation of immorality and evil (Nurse Ratchet, Dr Evil, Patrick Bateman), a phenomenon encapsulating wickedness or harm (war, climate change, racism), or socially unacceptable or self-experienced struggles and traits (procrastination, over-eating, pettiness). Once again, this relates to the story elements of plot (Herman 2004; Ricoeur 1980) and character (Bal 2009; Bates 1994), where events are given interconnected meaning that accumulates to explanatory factors. Our propensity to think in absolutes and to shy away from that which challenges our worldview reverberates in the creation of echo chambers (Edwards 2013) where fear can be faced though shared perceptions of good and evil. They can also increase fear of ‘the outside’, the arenas not part of your echo chamber where other, potentially challenging, experiences and beliefs test the strength of your own convictions.

Echo chambers have played a central role during the COVID-19 crisis. A global pandemic with deaths in the millions is bound to cause a great deal of fear, under the influence of which the lines between us and them become more pronounced, relying on processes and justifications of othering (de Beauvoir 1949; Riggins 1997), a process shown to be particularly prominent in responses to perceived threats such as infectious disease (Schaller and Neuberg 2012). Othering in the age of COVID has gotten some attention already (e.g. Reny and Barreto 2020), not least in its role in separating people according to race, socio-economic status, and healthcare access as discussed by White (2020) who highlights how xenophobic and racist outburst in relation to the pandemic mirrors historical reactions to pandemic disease threats where epidemics have been intimately linked to global commerce and exploitation. This othering operates both within nations and between them as it travel across the world along with the spread of the pandemic, starting with the other as China, then Italy, then the US, and now as mutated strains often known as the countries in which they were first found: UK, South Africa, Brazil, India. Partly as a response to this practice, the so called ‘variants of concern’ are now named after the Greek alphabet which serves to show the power of naming. Even more prominently, othering is connected to national responses to the pandemic. Sweden, a country usually of little international interest, has taken to the world stage as either ‘a future model’ (Waterfield and Tang 2020) or as a plague riddled madhouse that not even its neighbours will touch (Henlay 2020). Sweden’s approach to the pandemic has also led to a prime example of how storytelling can mix opposing beliefs and values into the same plot, a process Barthes proposes to be reliant on neologism, or transitory concepts related to specific contingencies (Barthes 1972, 1975). The word ‘plandemic’, a combination of plan and pandemic to which we will return later, could be such a neologism, should it gain traction, or new meanings to old words such as ‘freedom’, which shifts between prepositions—from, to, in—and across geographies and time to hold diametrically opposing meanings. In this way, stories become worlds unto themselves, requiring internal logic—‘that makes sense to me’—but not necessarily external validation, which enables cherry-picking and interpreting events to fit a plot. In the example of Sweden, neologism and creating internal logics can be seen in the rhetoric of American right-wing populists for whom Sweden has been a long-standing example of the socialist (or communist) nightmare, from the days of Olof Palme (Marklund 2016) to Trump’s infamous ‘last night in Sweden’ remarks (Chan 2017). In the American re-open rallies we could see signs saying ‘Legalise the Constitution’ and ‘Freedom over safety’ alongside signs telling the US to ‘Be like Sweden’ (Stopera 2020). And yet the rationales and justifications for the strategic response in Sweden—trust in the state and a strong social contract—are fundamentally opposed to American right-wing conservatism where distrust in the state is widespread and individual achievement is hailed over collective approaches. It is also worth noting that the biggest domestic critics of Sweden’s approach are the Sweden Democrats, an opposition party on the far-right. These are oppositions about which stories and storytelling do not care and a case of making meaning and events change to fit the plot.

Similar use of fear, and in some cases fearmongering, can be seen in naming the villain, where several attempts have been made at infusing the name of the virus itself with blame, e.g. ‘the Wuhan flu’, ‘the Chinese flu’, or ‘Winnie the Flu’, the last being named from a long-riding joke deriding the Chinese president Xi Jinping. While often humorous in intent, this naming nevertheless colours our impression of events and actions relating to ‘the antagonist’ and can make us more susceptible to new events and actions that confirm the character and plot, e.g. choosing to focus on how China has contributed medical equipment around the world (Hansrod 2020), or how the donations may be part of a political ruse to create tension or access (Wong and Paul 2020), or how some of the equipment has been faulty (BBC 2020a). What we are struggling with here is *complexity*, or rather a lack thereof, which brings us full circle back to one of storytelling’s most valuable assets and greatest flaws, i.e. dealing with fear through simplification.

In sum, storytelling is one of our most central and effective ways of coping with fear through acts of simplification, the naming of the villain, and the juxtaposition of hope presented as a clear and effective course (plot) of action. For processes of transformation, fear is a balance act, a set of scales that oscillate between constructive alarm and anxiety-induced lethargy. Not least, *the spread* of fear and/or hope is of central importance to processes of transformation. Here, technological developments have been crucial in increasing story proliferation, leading to new narrative practices, and potentially even a new form of storyteller: the algorithmic.

### Digitalisation

Alongside the rise, spread, and development of the Internet, storytelling has become an increasingly digital affair. The pandemic has served to further increase this trend as traditional public places of political discourse (Habermas 1989) close down and social distancing cuts many of us off from all but our closest family members. It would be a mistake to consider this digitalisation of storytelling to be of an exclusively or even predominately technical nature. Rather, online storytelling entails several qualitative differences. This paper will address two such differences: the sharing storyteller and non-human actors.

Online storytelling is mainly self-narrated, i.e. stories about oneself by oneself via blogs, Facebook updates, tweets, or emails (Khosrow-Pour 2019), as well as highly fragmented, consisting mainly of ‘narbs’ or ‘a small narrative bit (…) that tells a tiny story about an individual’ (Mitra 2010, p. 4). These short autobiographical stories are often transformative in style, what Barros (1998) calls ‘narratives of transformation’. They centre on the notion of metamorphoses, showing how events, defined and emplotted, are direct causes of change. As such, they are used to explain and convey the character *I*, i.e. I am Y because of X. Or rather, I am Y because of A–X, which will then explain why I do or act like Z. In many ways, this is simply a virtual version of face-to-face storytelling, where emplotted stories about ourselves and others are created and told to make sense of who we are and of the world we live in. Online, however, a large degree of peer-to-peer storytelling is performed via various forms of ‘shares’, which comes with a whole set of storytelling implications, including the proliferation of doubtful, uninformed, or full-out false information. False news may reach up to 100 times more people than true news (Vosoughi et al. 2018) and the number of studies showing our propensity to share false news, even knowingly, is growing (Vicario et al. 2016; Vosoughi et al. 2018). These shares are not necessarily motivated by ignorance or a desire to spread misinformation, but may equally be driven by a desire to amuse, i.e. sharing an outrageous tweet, blog, or newspaper article with a friend to get a laugh. In addition, there is rising evidence that many of these shares are ‘silent’, i.e. sharing a link without first clicking it yourself. Gabielkov et al (2016) found that as many as 59% of all URLs shared on Twitter are not visited before being shared, although we should note that this only applies to actions within Twitter and that the URLs could have been accessed inside the source domain or via a search engine. Similarly, but less scientifically rigidly, an article in *The Science Post* entitled ‘Study: 70% of Facebook users only read the headline of science stories before commenting’ (The Science Post 2018), has been shared almost 130,000 times, even though it consists almost exclusively of an extended Ipsum Lorem text. Many of these shares will be motivated by humour and sarcasm, but far from all and potentially with the same end result in terms of story proliferation due to difficulties in conveying intent, such as sarcasm, online. Digital storytelling lacks many important communication signifiers that convey intent, such as facial expression, tone, and cadence. Some of these missed markers are compensated for using symbols such as emojis (consider the difference between ‘interesting article’ and ‘interesting article!! ’), but these are personal preferences, the meaning of which can easily be lost or confused. Even more to the point, we must ask the harm in sarcastic sharing even when the sarcasm is successfully conveyed as repetition may still serve to naturalise the story (Barthes 1972) even when laughed at. In addition, the social nature of online sites may itself be a complicating factor as we are less inclined to question statements when having to do so in the (virtual) presence of others (Jun et al. 2017). These tendencies are not unfamiliar in the non-digital storyteller, but the speed, quantity, and culture of online interaction has nevertheless given rise to a type of storyteller, the sharing storyteller.

These issues are further complicated by the second point, the involvement and influence of non-human actors in the form of algorithms created to collect, analyse, and interact with online users and their data. Social bots (Ferrara et al. 2016) are becoming increasingly proficient in manipulating online conversation across social platforms, excelling in preforming one of the most central practices of story naturalisation, i.e. repetition (Barthes 1972). While mostly connected to the narrative type known as myth, repetition is crucial to all storytelling as it creates familiarity and acceptance as repeating a story over and over achieves authority by recognition (Hall 2006). Bots are capable of spreading a story both within and across platforms at remarkable speed and rate, creating the illusion of general acceptance or ‘common sense’. In addition to this active participation in storytelling, about which we need to know a great deal more, algorithms are themselves being *storytold*. The leading character here is ‘the Bot’, which is part of plots designed to create fear to achieve political or financial gain or influence. But like most story characters, bots are neither bad nor good by nature, but can *do* bad or good depending on context as well as point of view.

Both digitalisation and automatisation can be illustrated by COVID-19 storytelling, not least in terms of a marked increase in the level of bot activity. A research team at Carnegie Mellon University found that while bot involvement in major events typically lies around 10–20%, of the more than 200 million tweets discussing coronavirus or COVID-19 they collected, 82% of the 50 most influential retweeters were bots (Young 2020). These automated programs are capable of a wide range of online functions, including engaging in conversations and rebroadcasting messages repeatedly and across platforms. Many of these bots and cyborgs, the latter being accounts run by both bots and humans, were created in February of 2020 and have become both more sophisticated, e.g. engaging in deep networking, and more directed, e.g. targeting minority or risk groups (Young 2020).

In terms of the kind of COVID-19 stories that are being told online, one of the persisting and, at the moment of writing still highly controversial, COVID-19 storylines is that of re-opening or returning to normal. This storytelling focuses not only on human and societal capacity, weighing risks and rewards of each choice against each other, but on political allegiances that influence and sometimes override scientific data, not least in the US (Bruine de Bruin et al. 2020; Kreps and Kriner 2020), where re-opening movements are taking to the keyboards as well as the streets in an effort to make their voices heard and demands met. The result has been a confused type of storytelling, where plots, timelines, and characters are intermingled with a jumble of political messaging. Examples include the adoption and adaptation of conspiracy theories, such as in the short film Plandemic released on social media platforms on 4 May, 2020, in which former medical researcher Judy Mikovits makes several wide claims, including facemasks making you sick by ‘activating’ viruses. The video was taken down by several platforms and its claims were swiftly debunked (Enserink and Cohen 2020), but the speed and spread of online storytelling made such efforts near pointless. The video was viewed more than 8 million times in the first week (Alba 2020) and in the video, viewers are actively urged to share it on other platforms ‘in an effort to bypass the gatekeepers of free speech’ (BBC 2020b). Here, we see the sharing storyteller in action, using the speed, often in combination with the automatisation, of the web to proliferate and naturalise storylines across platforms and audiences.

These examples illustrate the argument that digital storytelling may differ both qualitatively and quantitatively from traditional storytelling in ways that greatly limit the potential of co-creative storytelling, where participants typically number in the single digits, processes are comparatively slow, and most storytellers are human.

## Discussion: COVID-19 & climate change

As two global and urgent phenomena requiring immediate and collective action, it is tempting to draw parallels between the COVID-19 pandemic and climate change. Comparisons between the two crises have been made throughout the pandemic, focusing primarily on either drawing lessons from the COVID-19 pandemic or considering the pandemic an opportunity or initiator for climate change adaptation or mitigation measures (e.g. Bertram et al. 2021; Bouman et al. 2020; Klenert et al. 2020; Manzanedo and Manning 2020). But while it is argued that ‘the climate emergency is like the COVID-19 emergency, just in slow motion and much graver’ (Hepburn et al. 2020, p. 359), narratively, the two show fundamental differences that warn against transferring storytelling practices and knowledges from one crisis to the other. Note that neither COVID-19 nor climate change are in any way singular, universal, or consistent stories, but a myriad of stories, often fragmented, that change across time, cultures, geographies, societies, and between and within individuals. Hence, this section does not aim to identify any core narrative, but focuses on broad and overarching characteristics with the aim to illustrate the importance of story elements.

First, the two crises differ in aspects of time (Ricoeur 1984, 1985, 1988). Chronologically, both pandemic and climate change stories follow a basic plot-line of one event leading to another, but the difference in timescale creates a dissimilar notion of the ‘now’ or the middle. COVID-19 is here and now while climate change can easier be perceived of as there and then, in the past and future. Reconnecting to the crucial element of a timeline, climate change stretches over centuries, both forward and backwards, which gives the illusion of either (i) being irrelevant to our daily lives or (ii) being locked-in in deterministic trajectories from which it is already too late to diverge. For storytelling, this can translate into beginning and resolution being perceived of as disconnected, too far apart. In narrative terms, this disconnect challenges the story structure of a beginning, a middle, and an end, creating fragmentation or incoherence as well as a reduced chance of engagement as it does not provide narrative direction. Placing events on a timeline is itself difficult as overarching, intangible, and comparatively slow changes (global warming) are offset by immediate and subjective experiences (a cold winter). Physical changes pose similar challenges as climate change impacts are either slow by human standards, i.e. slow-onset events (UNFCCC 2012), or immediate but results of cumulative processes that blur the narrative timeline. Thorny interconnections are required, forcing the story to deal with a complexity it is meant to reduce, not embrace. Attempts have been made to counteract timeline fragmentation, such as by Pileggi and Lamia (2020) who used Ontology Web Language to collect, classify, and connect climate change events to ‘tell the climate change story so far’ (Pileggi and Lamia 2020, p. 65,294). The sheer scale, complexity, and potential controversy of such a project, using Semantic Web technology to identify and classify climate change-related events from multiple perspectives, show the challenge of creating a climate change timeline, let alone a coherent story.

In addition to the time aspect, fear plays a different role in the two crises, particularly in how it effects the act of naming the enemy. COVID-19 is a clearly demarcated antagonist, the fear of which urges a clear course of action. The comparative ease of naming the antagonist is to a large extent related to fast and measurable consequences, i.e. cases and deaths, compared to which the impacts of climate change are relatively abstract. If deaths connected to climate change, such as heat stress and malaria, were to be spectacular, e.g. through self-combustion, rather than creeping and ambiguous, the antagonist may have been more easily defined. Had this been the case, air pollution alone would have caused as many as 9 million people to go up in flames in 2015 (Lelieveld et al. 2019). If, in addition, climate change-related illnesses, such as asthma, stunted growth, malnutrition, lower cognitive capacity, were to be officially diagnosed as a ‘climate change illness’, the threat would quickly go from abstract to real. As is, the antagonist remains difficult to define and combat. In addition, the villain’s name—climate change—is in itself more contested than that of COVID-19, complicating the crucial act of naming the enemy needed for a narrative relief of anxiety and fear (Cassirer 1946). ‘Name changes’, such as that from global warming to climate change, can be framed as either the villain having been misidentified (scientifically unsound) or as being a result of political interests (Jang and Hart 2015; Villar and Krosnick 2011). While climate change does not suffer from a lack of evidence, it does suffer from a lack of narrative clarity.

For storytelling aiming at creating constructive narrative processes of transformation, differences such as these are crucial. Transformative storytelling, then must engage with the complexity inherent not only in the sustainability challenge, but in the very nature of the story itself. If not, using storytelling as part of participatory and co-creative processes risks either not fulfilling its transformative potential or, at worst, missing its mark entirely and being dismissed as ineffective.

## Conclusion

This paper has put focus on the intersection of narrative approaches and the field of sustainability science and can, using the two crises of COVID-19 and climate change as illustrations, present three conclusions.

First, sustainability science is increasingly engaging with narrative concepts and practices centring on the transformative potential of storytelling. While both valid and valuable, such engagement needs to be sensitive to the fundamental messiness of storytelling. More specifically, if the transformative potential of storytelling is to be realised, there needs to be a better understanding of story elements—plot, fear/hope, time, character creation—and how they interplay to create new narratives and practices. Narrative deficiencies, such as an incomplete plot, an unnamed fear, or a vague timeline, severely limit narratives of transformation as they lack crucial elements needed to complete the full story. The specificities of story elements warn against one-to-one comparisons, broadly illustrated here by the crises of COVID-19 and climate change, and urge us to consider each story as custom-made rather than one-size-fits-all. In short, sustainability science must embrace its systemic foundations by considering *stories as a whole consisting of interconnected parts*.

Second, the story element of fear is crucial in crisis storytelling, both as part of the narrative process of naming the antagonist and as a basis for action as the story helps to alleviate anxiety by presenting a solution that incorporates plot, timeline, and character creation. However, if fear is to be a positive force, high efficacy is needed in the form of clear available action lest it lead to defensive and apathetic responses (Witte and Allen 2000). Climate change narratives are particularly sensitive to this risk, combining a high level of fear and, due to its (perceived) complexities and comparatively vague antagonist, low efficacy. In comparison, COVID-19 narratives also comprise high levels of fear, but these are better matched with a well-defined antagonist and a clear plot, i.e. the ability to alleviate anxiety by naming a common enemy and the way in which to conquer it. Time or timeline plays an important role here, comparing the fast and clear impacts of COVID-19 (cases and deaths) to the protracted and cumulative effects of climate change. Thus, from a story perspective, sustainability science needs to find ways to acknowledge and often even raise fears while also finding and engaging in transformative practices that demonstrate and narrate *the ability to effect change in the face of fear*.

Third, the digitalisation and automatisation of storytelling is not merely telling stories on a new platform, but a development that deeply affects how and to whom we tell stories as well as who (or what) tells them. Digitalisation has created a new storyteller, ‘the sharing storyteller’, which tells stories using various forms of shares across predominately social platforms. This type of storytelling is fast, often autobiographical, and metamorphic, i.e. short stories about oneself that explain the self, and it is lacking in its verification as many shares are blind, i.e. shared without ever having been read. Furthermore, non-human actors in the form of machine learning algorithms collect, interact with, and employ user data to find and spread stories using advanced, yet to the audience hidden, predictive capabilities. Yet, engaging with algorithmic practices from a narrative perspective is not a matter of challenging the spread of misinformation, but of considering how algorithmic practices tell, create, and naturalise stories, regardless of their factual accuracy as the opposition between stories and truth is both false and counterproductive. Not even one of the most ardent, entrenched, and stubborn of narratives—the myth (Essebo 2019)—is by nature contradictory to ‘truth’, but rather operates *irrespective* of it, relying on processes of naturalisation and societal assimilation. Not least, this false binary prevents us from engaging with climate change denial as our only approach would be presenting ever more facts to an audience that has already decided against these facts ad infinitum. Misinformation must be challenged, but doing so includes understanding it for its narrative purpose and use. Alternative facts need to be met with alternative stories, even more so in the digital era when the algorithmic sharing storyteller can spread stories far faster than anyone can fact check, let alone refute. Looking forward, both sustainability science and narrative research need to engage with algorithmic practices that alter and create novel storytelling practices as well as new combinations of non-human/human hybrid storytellers. *Engaging with algorithmic storytelling practices* is one of sustainability science’s most urgent tasks*.*

Storytelling, as a practice that inspires, creates, and legitimises change, is vital for transformational processes. In engaging deeper with storytelling, sustainability science could not only learn to better understand the fear, confusion, and misperceptions associated with vast and overwhelming environmental challenges, but to use the creative power of storytelling in creating pathways forward. Doing so will require acknowledging the inherent complexity of stories, realising the role and potential of fear, and engaging in rapidly evolving algorithmic storytelling practices and technologies. In short, it would require truly exploring the transformative potential of stories.
